# Risk of Chronic Kidney Disease in Pneumoconiosis: Results from a Retrospective Cohort Study (2008–2019)

**DOI:** 10.3390/biomedicines11010150

**Published:** 2023-01-06

**Authors:** Chao-Hsuan Wei, Chia-Hsiang Li, Te-Chun Shen, Yu-Tung Hung, Chih-Yen Tu, Te-Chun Hsia, Wu-Huei Hsu, Chung Y. Hsu

**Affiliations:** 1 Division of Nephology, Department of Internal Medicine, An Nan Hospital, China Medical University, Tainan 709, Taiwan; 2 Division of Pulmonary and Critical Care Medicine, Department of Internal Medicine, China Medical University Hospital, Taichung 404, Taiwan; 3 School of Medicine, College of Medicine, China Medical University, Taichung 404, Taiwan; 4 Division of Critical Care Medicine, Chu Shang Show Chwan Hospital, Nantou 557, Taiwan; 5 Management Office for Health Data, China Medical University Hospital, Taichung 404, Taiwan

**Keywords:** interstitial lung disease (ILD), pneumoconiosis, chronic kidney disease (CKD)

## Abstract

Background: Pneumoconiosis has considerable comorbidities, most notably pulmonary and cardiovascular diseases. However, the relationship between pneumoconiosis and chronic kidney disease (CKD) is largely unknown. The present study aimed to use a retrospective cohort study design to further clarify the association between pneumoconiosis and subsequent CKD risk. Methods: This is a nationwide, population-based, retrospective cohort study that used data from Taiwan’s National Health Insurance Database. Between 2008 and 2018, 17,952 newly diagnosed patients were included in the pneumoconiosis cohort, while 71,808 individuals without pneumoconiosis were included in the comparison cohort, with a propensity score matching for age, gender, and date of pneumoconiosis diagnosis. The development of CKD was monitored until the end of 2019. The risk was assessed using Cox proportional hazard regression models. Results: After controlling for age, gender, and comorbidity, the overall incidence of CKD was 1.69-fold higher in the pneumoconiosis cohort than in the comparison cohort (19.71 vs. 11.76 per 1000 person-years, respectively, *p* < 0.001), with an adjusted hazard ratio of 1.83 (95% confidence interval: 1.73–1.93). Stratified analyses by age group, gender, and presence of comorbidity revealed that the adjusted hazard ratios of CKD associated with pneumoconiosis remained significant (8/9). Furthermore, pneumoconiosis and tri-high (hypertension, hyperglycemia, and hyperlipidemia) interact positively with CKD development (*p* < 0.001). Conclusion: Patients with pneumoconiosis had a significantly higher risk of developing CKD than those without. Pneumoconiosis combined with hypertension, hyperglycemia, or hyperlipidemia would increase the risk even further. More studies are required to understand the possible pathophysiological mechanisms.

## 1. Introduction

Pneumoconiosis is a group of heterogeneous occupational interstitial lung diseases caused by the inhalation of mineral dust in the lungs [1]. It is prevalent worldwide, and there has remained a high incidence in recent years. According to a systematic analysis for the Global Burden of Disease Study, the prevalence of pneumoconiosis is estimated to be around 527,500 cases, with over 60,000 new cases reported each year. Since 2015, pneumoconiosis mortality has remained high, with over 21,000 deaths each year [2]. Mineral dust is a complex mixture that can cause a wide range of respiratory and nonrespiratory diseases. Exposure to mineral dust is associated with increased mortality from these diseases [3,4,5].

Pneumoconiosis has been linked to several comorbidities, particularly pulmonary and cardiovascular complications [6,7,8,9,10]. Common comorbidities include hypertension, chronic obstructive pulmonary disease (COPD), hyperlipidemia, diabetes mellitus (DM), ischemic heart disease (IHD), atrial fibrillation, congestive heart failure, gastroesophageal reflux disease, benign prostatic hyperplasia, chronic kidney disease (CKD), and hypothyroidism [5,11]. Among these, CKD is a reasonable but largely unexplored comorbidity.

Pneumoconiosis could be subdivided into several categories, such as coal workers’ pneumoconiosis, asbestosis, and silicosis. Silicosis has been reported to have an association with CKD [12,13,14]. Previous studies suggest that either a direct toxic action of inhaled silica on the glomerulus and proximal tubules or other autoimmune processes, such as silica-induced scleroderma, lead to chronic kidney injury. In addition, dust particles (<2.5 µm in diameter) can be inhaled deeply into the lungs, with a portion deposited in the alveoli and entering the pulmonary and systemic circulation, leading to an inflammatory response, vascular injury, and thromboembolic processes, which can contribute to the development of cardiovascular disease and CKD [6,15,16]. However, the precise association between pneumoconiosis and CKD remains inconclusive. This study aimed to further clarify the association between pneumoconiosis and the risk of developing CKD.

## 2. Materials and Methods

### 2.1. Data Source

Taiwan’s National Health Insurance (NHI) program was established in 1995 and has covered 99.5% of its residents or more since 2010. The National Health Insurance Database was set up and is managed by Taiwan’s Ministry of Health and Welfare. The National Health Insurance Database, which included 31,486,225 people and detailed medical information from 2008 to 2019, was used in this study. This study was approved by the Research Ethics Committee of the China Medical University Hospital (CMUH110-REC3-133).

### 2.2. Study Cohorts

**Figure 1:**
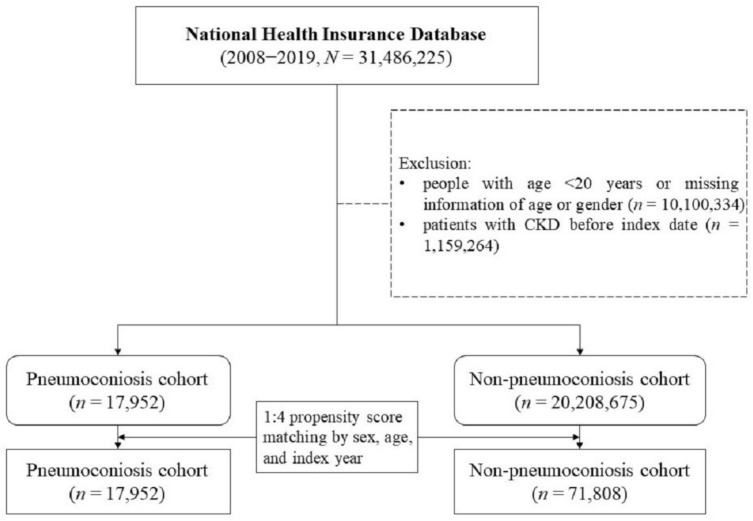
Flowchart of participants selection.

People under 20 years old were excluded from the study because they have less work experience and insufficient dust exposure to develop pneumoconiosis. Adults newly diagnosed with pneumoconiosis (International Classification of Diseases [ICD] codes 500–505 and J60–J65) between 2008 and 2018 were included in the pneumoconiosis cohort (Figure 1). Similar methods have been reported elsewhere [9]. Patients who had CKD prior to the diagnosis of pneumoconiosis were excluded. Adults without pneumoconiosis were included in the comparison cohort, with a propensity score matching for age, gender, and date of pneumoconiosis diagnosis. Both cohorts were followed until a new CKD diagnosis, insurance system withdrawal, death, or the end of 2019, whichever came first.

### 2.3. Outcome and Covariates

In this study, the primary outcome was CKD (ICD codes 585 and N18.4, N18.5, N18.6, and N18.9), with age, gender, and comorbidities acting as confounding factors. Therefore, several related comorbidities at baseline were selected for adjustment, including hypertension (ICD codes 401–405 and I10–I16), DM (ICD codes 250 and E08–E13), hyperlipidemia (ICD codes 272 and E78), IHD (ICD codes 410–414 and I20–I25), and cerebrovascular disease (CVD, ICD 430–438 and I60–I69).

### 2.4. Statistical Analysis

The proportional distributions of age group, gender, and comorbidity were compared between the two cohorts using the Chi-squared test, while the mean age was compared using the Student’s *t*-test. In addition, we calculated standardized mean difference (SMD) to compare the differences of categorical and continuous variables between the two cohorts. The cumulative incidence of CKD in the pneumoconiosis and comparison cohorts was estimated using the Kaplan–Meier method, with the log-rank test used to determine significance. Univariable and multivariable Cox proportional hazard regression models were used to estimate crude and adjusted hazard ratios (cHRs and aHRs), as well as 95% confidence intervals (CIs). Data were analyzed using the SAS statistical software (version 9.4 for Windows; SAS Institute, Inc., Cary, NC, USA). A *p*-value of less than 0.05 was considered statistically significant.

## 3. Results

**Figure 2:**
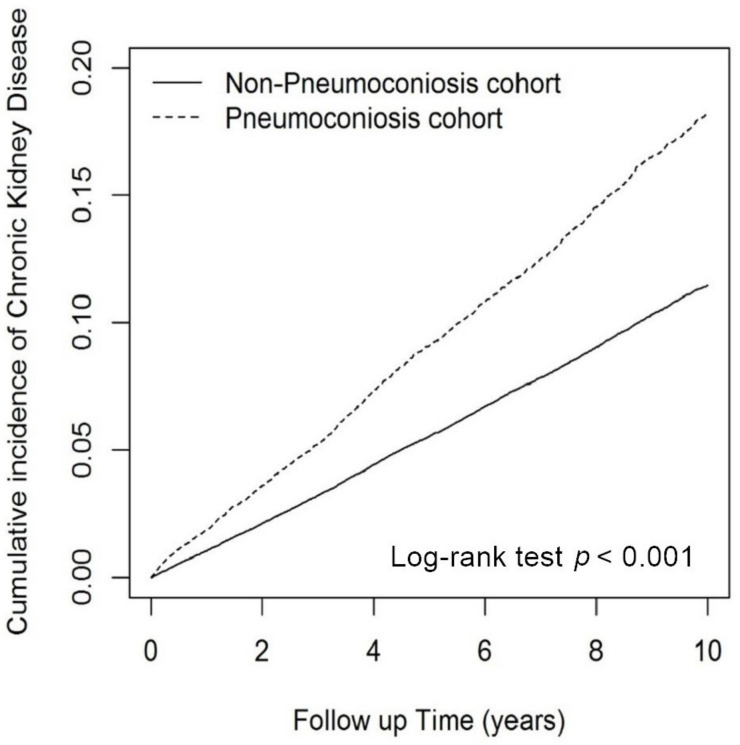
Cumulative incidence of chronic kidney disease in the pneumoconiosis and the nonpneumoconiosis cohorts.

This study included 17,952 patients in the pneumoconiosis cohort and 71,808 individuals in the comparison cohort (Table 1). The mean age was 69.67 ± 11.45 years in the pneumoconiosis cohort and 69.62 ± 11.62 years in the comparison cohort. The pneumoconiosis cohort had 87.43% males, while the comparison cohort had 87.49% males. The proportions of comorbidities in the pneumoconiosis and comparison cohorts were as follows: hypertension (37.73% vs. 34.09%); hyperlipidemia (19.35% vs. 19.17%); IHD (15.84% vs. 12.68%); DM (15.02% vs. 15.99%); and CVD (11.56% vs. 9.23%). The mean follow-up time was 5.43 ± 3.66 and 7.11 ± 3.60 years in the pneumoconiosis and comparison cohorts, respectively. During the follow-up period, the cumulative incidence of CKD was significantly higher in the pneumoconiosis cohort than in the comparison cohort (Figure 2, *p* < 0.001 in the log-rank test).

The overall incidence of CKD was 1.69-fold higher in the pneumoconiosis cohort than that in the comparison cohort (19.71 vs. 11.76 per 1000 person-years, respectively, *p* <0.001, Table 2), with an aHR of 1.83 (95% CI: 1.73–1.93) after controlling for age, gender, and comorbidity. The aHRs of CKD were 2.43-fold higher in patients aged 50–64 (95% CI: 1.93–3.07) and 4.84-fold higher in those aged 65 or older (95% CI: 3.87–6.05) than in those aged 20–49. The aHR of CKD was 1.42-fold higher in men than in women (95% CI: 1.32–1.53). Furthermore, the CKD risk was significantly higher in patients with hypertension (aHR: 2.76, 95% CI: 2.62–2.91), DM (aHR: 2.07, 95% CI: 1.96–2.19), IHD (aHR: 1.24, 95% CI: 1.17–1.31), CVD (aHR: 1.19, 95% CI: 1.12–1.27), and hyperlipidemia (aHR: 1.08, 95% CI: 1.03–1.15) than in those without these comorbidities.

After adjusting for age, gender, and the presence of comorbidities, the incidences and aHRs of CKD in the pneumoconiosis cohort were all significantly higher than in the comparison cohort (Table 3). In the 20–49, 50–64, and ≥ 65 age groups, the age-specific aHRs in the pneumoconiosis cohort compared to the comparison cohort were 1.35 (95% CI: 0.79–2.32), 1.85 (95% CI: 1.59–2.14), and 1.85 (95% CI: 1.75–1.96), respectively. The gender-specific aHRs in the pneumoconiosis cohort compared to the comparison cohort were 1.96 (95% CI: 1.66–2.30) in females and 1.82 (95% CI: 1.72–1.92) in males. The comorbidity-specific aHRs in the pneumoconiosis cohort compared to the comparison cohort were 2.07 (95% CI: 1.94–2.21) in those with comorbidity and 1.22 (95% CI: 1.11–1.34) in those without.

Table 4 shows the risk of CKD in pneumoconiosis in relation to tri-high (hypertension, hyperglycemia, and hyperlipidemia). The aHRs of CKD in those with any one of the tri-highs but no pneumoconiosis, those with pneumoconiosis but no tri-high, and those with both pneumoconiosis and any one of the tri-highs were 4.53 (95% CI: 4.21–4.87), 3.97 (95% CI: 3.63–4.33), and 5.01 (95% CI: 4.50–5.58), respectively, compared to those with neither pneumoconiosis nor tri-high, with a significant *p*-value for the interaction (*p* < 0.001). Similar results were obtained when pneumoconiosis interacted with any two of the tri-high (*p* < 0.001) and all the tri-highs (*p* < 0.001).

## 4. Discussion

In the nationwide, population-based, retrospective cohort study evaluating the relationship between pneumoconiosis and subsequent CKD risk, patients with pneumoconiosis had a significantly higher risk of CKD than those without. Further analysis showed that pneumoconiosis-associated CKD risk was significantly higher in most age, gender, and comorbidity subgroups. Furthermore, pneumoconiosis and tri-high (hypertension, hyperglycemia, and hyperlipidemia) interact positively with CKD development. Finally, the study provided valuable epidemiologic data for pneumoconiosis on a large scale.

The prevalence of CKD in patients with pneumoconiosis has been reported. Between 2011 and 2014, Paul et al. collected data on 8531 patients with pneumoconiosis from the 5% Medicare Claims Limited Data Set in the United States, and 2568 patients died during the follow-up period [5]. They reported that CKD accounted for 6.8% of all patients with pneumoconiosis and 12.5% of those who died. In another study, Hu et al. collected 12,209 patients with pneumoconiosis from the Taiwan National Health Insurance Database and reported that 535 (4.38%) of them had CKD [9]. This indicates that CKD is a significant comorbidity of pneumoconiosis.

Only a few studies have directly focused on pneumoconiosis and the subsequent development of CKD, and these studies all investigated exposure to silica [12,13,14,17,18]. Rapiti et al. [12] first reported that ceramic workers exposed to silica dust develop end-stage renal disease more frequently than those who are unexposed. Rosenman et al. [13] reported that approximately 58 of 583 patients with silicosis had CKD. Millerick-May et al. [14] reported that approximately 740 of 1072 patients with silicosis had stage I or greater CKD. However, Steenland et al. [17] did not find a significant association between silicosis and end-stage renal disease. Additionally, Raanan et al. [18] did not identify an association between occupational silica exposure and renal disease. In the present study, the risk of CKD was significantly higher in patients with silicosis than those without pneumoconiosis (13.93 vs. 11.76 per 1000 person-years, aHR: 1.32, 95% CI: 1.03–1.69).

The underlying mechanism of the association between pneumoconiosis and CKD has largely remained unknown. The most common causes of CKD are DM and hypertension. Hyperglycemia primarily causes increases in growth factor, angiotensin II, endothelin, and advanced glycation end products, which contribute to hyperfiltration, whereas hypertension primarily causes abnormal autoregulation of the afferent arteriole, leading to hyperfiltration [19]. Pneumoconiosis-related inflammatory responses, vascular injury, and thromboembolic processes may all play a role in the development of CKD [6]. Furthermore, previous studies on silicosis and CKD suggested that either a direct toxic action of inhaled dust or other autoimmune processes could lead to chronic kidney injury [12,13,14].

The risk of CKD in the different categories of pneumoconiosis is unknown. In this study, the incidence of CKD was 21.70 per 1000 person-years in those exposed to coal dust, 17.48 per 1000 person-years in those exposed to asbestos, and 13.93 per 1000 person-years in those exposed to silica. Interestingly, patients with coal workers’ pneumoconiosis had a higher incidence of CKD than those with other categories of pneumoconiosis, suggesting that further investigation is necessary. We also found a typical dose-dependence of the tri-high on the CKD risk in patients with pneumoconiosis. We must pay more attention to the development of CKD in pneumoconiosis patients with additional tri-high comorbidities.

The strength of this study is the establishment of a large-scale pneumoconiosis cohort and a well-matched cohort for comparison. Almost all participants completed the follow-up. A prospective cohort study is costly; thus, a retrospective cohort study using Taiwan NHIRD is a suitable and economical alternative [20,21]. Furthermore, this study was able to reflect a “real-world” scenario in which pneumoconiosis, CKD, and all comorbidities were directly diagnosed during medical consultation.

Our study has some limitations that should be considered. First, pneumoconiosis, CKD, and comorbidities were defined using ICD codes, and all diagnoses were based on clinical physicians’ competence. Second, the database lacks detailed information on smoking habits, occupational exposure, and family history, all of which are related factors. Third, relevant clinical variables, such as laboratory data, pulmonary function tests, imaging results, and pathology findings, were unavailable.

## 5. Conclusions

Patients with pneumoconiosis had a significantly higher risk of developing CKD than those without. Pneumoconiosis combined with hypertension, hyperglycemia, or hyperlipidemia would increase the risk even further. More studies are required to understand the possible pathophysiological mechanisms.

## Figures and Tables

**Table 1:** Characteristics for individuals with and without pneumoconiosis.

	Pneumoconiosis				*p*-Value ^†^	SMD ^#^
	No		Yes			
	N = 71,808		N = 17,952			
	*n*	*%*	*n*	%		
Age					0.9942	0.002
20−49	4165	5.80	1045	5.82		
50−64	15,600	21.72	3900	21.72		
≥65	52,043	72.48	13,007	72.45		
Mean ± SD	69.62	±11.62	69.67	±11.45	0.6080	0.001
Gender					0.8323	<0.001
Men	62,826	87.49	15,696	87.43		<0.001
Women	8982	12.51	2256	12.57		0.004
Comorbidity						
Hypertension	24,482	34.09	6774	37.73	<0.001	0.076
Diabetes mellitus	11,481	15.99	2696	15.02	0.0014	0.027
Hyperlipidemia	13,764	19.17	3473	19.35	0.5876	0.005
Ischemic heart disease	9103	12.68	2844	15.84	<0.001	0.091
Cerebrovascular disease	6628	9.23	2076	11.56	<0.001	0.077

SD, standard deviation; SMD, standardized mean difference. ^†^ Chi-squared test and *t*-test. ^#^ A SMD < 0.1 indicates a negligible difference between the two cohorts.

**Table 2:** Risk factor analyses for chronic kidney disease among all study individuals.

	Event	PY	IR ^†^	Crude HR (95% CI)	Adjusted HR ^#^ (95% CI)
Pneumoconiosis					
No	6003	510,372	11.76	1.00 (Reference)	1.00 (Reference)
Yes, overall	1922	97,496	19.71	1.69 (1.61−1.78) ***	1.83 (1.73−1.93) ***
Coal exposure	810	37,320	21.70	1.86 (1.73−2.00) ***	1.96 (1.82−2.11) ***
Asbestos exposure	25	1430	17.48	1.51 (1.02−2.24) *	1.24 (0.84−1.84)
Silica exposure	64	4595	13.93	1.20 (0.93−1.53)	1.32 (1.03−1.69) *
Age					
20−49	78	35,767	2.18	1.00 (Reference)	1.00 (Reference)
50−64	958	127,259	7.53	3.46 (2.74−4.35) ***	2.43 (1.93−3.07) ***
≥ 65	6889	444,842	15.49	7.11 (5.69−8.88) ***	4.84 (3.87−6.05) ***
Gender					
Women	751	72,305	10.39	1.00 (Reference)	1.00 (Reference)
Men	7174	535,563	13.40	1.29 (1.19−1.39) ***	1.42 (1.32−1.53) ***
Comorbidity					
Hypertension					
No	3204	433,138	7.40	1.00 (Reference)	1.00 (Reference)
Yes	4721	174,730	27.02	3.74 (3.58−3.92) ***	2.76 (2.62−2.91) ***
Diabetes mellitus					
No	5426	533,436	10.17	1.00 (Reference)	1.00 (Reference)
Yes	2499	74,431	33.57	3.37 (3.21−3.53) ***	2.07 (1.96−2.19) ***
Hyperlipidemia					
No	5690	514,953	11.05	1.00 (Reference)	1.00 (Reference)
Yes	2235	92,914	24.05	2.22 (2.11−2.33) ***	1.08 (1.03−1.15) ***
IHD					
No	6183	544,849	11.35	1.00 (Reference)	1.00 (Reference)
Yes	1742	63,019	27.64	2.47 (2.34−2.61) ***	1.24 (1.17−1.31) ***
CVD					
No	6740	566,416	11.90	1.00 (Reference)	1.00 (Reference)
Yes	1185	41,452	28.59	2.45 (2.30−2.60) ***	1.19 (1.12−1.27) ***

CI, confidence interval; CVD, cerebrovascular disease; IHD, ischemic heart disease; IR, incidence rate; HR, hazard ratio; PY, person-years. ^†^ Incidence rate per 1000 person-years. ^#^ Multivariable analysis including age, gender, and comorbidity. * *p* < 0.05; *** *p* < 0.001.

**Table 3:** Incidence rates and hazard ratios of chronic kidney disease for individuals with and without pneumoconiosis by age, gender, and comorbidity.

	Pneumoconiosis							
	No			Yes				
	Event	PY	IR ^†^	Event	PY	IR ^†^	Crude HR (95% CI)	Adjusted HR ^#^ (95% CI)
Age								Mode 1
20−49	59	29,070	2.03	19	6697	2.84	1.39 (0.83–2.33)	1.35 (0.79–2.32)
50−64	700	104,504	6.70	258	22,755	11.34	1.71 (1.49–1.98) ***	1.85 (1.59–2.14) ***
≥65	5244	376,798	13.92	1645	68,044	24.18	1.75 (1.65–1.85) ***	1.85 (1.75–1.96) ***
Gender	536	59,437		215	12,868			Mode 2
Women			9.02			16.71	1.87 (1.60–2.19) ***	1.96 (1.66–2.30) ***
Men	5467	450,935	12.12	1707	84,628	20.17	1.68 (1.59–1.77) ***	1.82 (1.72–1.92) ***
Comorbidity ^‡^								Mode 3
No	1436	324,304	4.43	807	54,453	14.82	3.48 (3.19–3.79) ***	3.60 (3.25–3.97) ***
Yes	4567	186,068	24.54	1115	43,043	25.90	1.07 (1.00–1.14) *	1.19 (1.11–1.27) ***

CI, confidence interval; HR, hazard ratio; IR, incidence rate; PY, person-years. ^†^ Incidence rate per 1000 person-years. ^#^ Multivariable analyses performed in mode 1 (adjusted for gender and comorbidity), mode 2 (adjusted for age and comorbidity), and mode 3 (adjusted for age and gender). ^‡^ Individuals with any comorbidity of hypertension, diabetes, hyperlipidemia, ischemic heart disease, or cerebrovascular disease were classified into the comorbidity group. * *p* < 0.05, *** *p* < 0.001.

**Table 4:** Cox proportional hazard regression analysis for the risk of chronic kidney disease-associated pneumoconiosis with interaction of tri-high (hypertension, hyperglycemia, and hyperlipidemia).

Variables		Case N	Event *n*	Adjusted HR ^†^ (95% CI)
Pneumoconiosis	1 of tri-high			
No	No	41,934	1601	1 (Reference)
No	Yes	14,801	1970	4.53 (4.21−4.87) ***
Yes	No	9711	871	3.97 (3.63−4.33) ***
Yes	Yes	4626	503	5.01 (4.50−5.58) ***
*p* for interaction < 0.001				
Pneumoconiosis	2 of tri-high			
No	No	41,934	1601	1 (Reference)
No	Yes	10,293	1588	6.04 (5.59−6.54) ***
Yes	No	9711	871	3.92 (3.59−4.29) ***
Yes	Yes	2508	370	7.68 (6.79−8.67) ***
*p* for interaction < 0.001				
Pneumoconiosis	3 of tri-high			
No	No	41,934	1601	1 (Reference)
No	Yes	4780	844	7.22 (6.54−7.97) ***
Yes	No	9711	871	3.67 (3.35−4.02) ***
Yes	Yes	1097	178	8.20 (6.93−9.70) ***
*p* for interaction < 0.001				

CI, confidence interval; HR, hazard ratio. ^†^ Multivariable analysis including age, gender, and comorbidity. *** *p* < 0.001.

## Data Availability

All data generated or analyzed during this study are included in this manuscript.

## References

[1] Qi X.M., Luo Y., Song M.Y., Liu Y., Shu T., Liu Y., Pang J.L., Wang J., Wang C. (2021). Pneumoconiosis: Current status and future prospects. Chin. Med. J..

[2] James S.L., Abate D., Abate K.H., Abay S.M., Abbafati C., Abbasi N., Abbastaber H., Abd-Allah F., Abdela J., Abdelalim A. (2018). Global, regional, and national incidence, prevalence, and years lived with disability for 354 diseases and injuries for 195 countries and territories, 1990–2017: A systematic analysis for the Global Burden of Disease Study 2017. Lancet.

[3] Hall N.B., Blackley D.J., Halldin C.N., Laney A.S. (2019). Current review of pneumoconiosis among US coal miners. Curr. Environ. Health Rep..

[4] Beggs J.A., Slavova S., Bunn T.L. (2015). Patterns of pneumoconiosis mortality in Kentucky: Analysis of death certificate data. Am. J. Ind. Med..

[5] Paul R., Adeyemi O., Arif A.A. (2022). Estimating mortality from coal workers’ pneumoconiosis among Medicare beneficiaries with pneumoconiosis using binary regressions for spatially sparse data. Am. J. Ind. Med..

[6] Shen C.H., Lin T.Y., Huang W.Y., Chen H.J., Kao C.H. (2015). Pneumoconiosis increases the risk of peripheral arterial disease: A nationwide population-based study. Medicine.

[7] Cheng Y.Y., Hsu K.H., Chen Y.H., Lin C.H. (2015). Increased risk of ischemic stroke in patients with pneumoconiosis. J. Clin. Neurosci..

[8] Chuang C.S., Ho S.C., Lin C.L., Lin M.C., Kao C.H. (2016). Risk of cerebrovascular events in pneumoconiosis patients: A population-based study, 1996–2011. Medicine.

[9] Hu W.S., Lin C.L. (2020). Risk of atrial fibrillation in patients with pneumoconiosis: A nationwide study in Taiwan. Clin. Cardiol..

[10] Yen C.M., Lin C.L., Lin M.C., Chen H.Y., Lu N.H., Kao C.H. (2016). Pneumoconiosis increases the risk of congestive heart failure: A nationwide population-based cohort study. Medicine.

[11] Sun B.Q., Zhao H.L., Xie Y. (2021). Progress in epidemiological studies on pneumoconiosis with comorbidities. Zhonghua Lao Dong Wei Sheng Zhi Ye Bing Za Zhi.

[12] Rapiti E., Sperati A., Miceli M., Forastiere F., Di Lallo D., Cavariani F., Goldsmith D.F., Perucci C.A. (1999). End stage renal disease among ceramic workers exposed to silica. Occup. Environ. Med..

[13] Rosenman K.D., Moore-Fuller M., Reilly M.J. (2000). Kidney disease and silicosis. Nephron.

[14] Millerick-May M.L., Schrauben S., Reilly M.J., Rosenman K.D. (2015). Silicosis and chronic renal disease. Am. J. Ind. Med..

[15] Nemmar A., Hoet P.H., Vanquickenborne B., Dinsdale D., Thomeer M., Hoylaerts M.F., Vanbilloen H., Mortelmans L., Nemery B. (2002). Passage of inhaled particles into the blood circulation in humans. Circulation.

[16] Sun Q., Hong X., Wold L.E. (2010). Cardiovascular effects of ambient particulate air pollution exposure. Circulation.

[17] Steenland K., Rosenman K., Socie E., Valiante D. (2002). Silicosis and end-stage renal disease. Scand. J. Work Environ. Health.

[18] Raanan R., Zack O., Ruben M., Perluk I., Moshe S. (2022). Occupational silica exposure and dose-response for related disorders-silicosis, pulmonary TB, AIDs and renal diseases: Results of a 15-year Israeli surveillance. Int. J. Environ. Res. Public Health.

[19] Charles C., Ferris A.H. (2020). Chronic Kidney Disease. Prim. Care.

[20] Hsing A.W., Ioannidis J.P. (2015). Nationwide population science: Lessons from the Taiwan National Health Insurance Research Database. JAMA Intern. Med..

[21] Lai S.W., Lin C.L., Liao K.F. (2017). Risk of contracting pneumonia among patients with predialysis chronic kidney disease: A population-based cohort study in Taiwan. Biomedicine.

